# Dengue Risk among Visitors to Hawaii during an Outbreak

**DOI:** 10.3201/eid1105.041064

**Published:** 2005-05

**Authors:** Carrie E. Smith, Tammy Tom, Jed Sasaki, Tracy Ayers, Paul V. Effler

**Affiliations:** * Hawaii State Department of Health, Honolulu, Hawaii, USA

**Keywords:** Arboviruses, dengue fever, emerging infectious diseases, hemorrhagic fever, travel medicine, vector-borne diseases

## Abstract

Despite the high rates of dengue in many tropical destinations frequented by tourists, limited information is available on the risk for infection among short-term visitors. We retrospectively surveyed 4,000 persons who arrived in Hawaii during the peak of the 2001–2002 dengue outbreak and collected follow-up serologic test results for those reporting denguelike illness. Of 3,064 visitors who responded, 94 (3%) experienced a denguelike illness either during their trip or within 14 days of departure; 34 of these persons were seen by a physician, and 2 were hospitalized. Twenty-seven visitors with denguelike illness provided a serum specimen; all specimens were negative for anti-dengue immunoglobulin G antibodies. The point estimate of dengue incidence was zero infections per 358 person-days of exposure with an upper 95% confidence limit of 3.0 cases per person-year. Thus, the risk for dengue infection for visitors to Hawaii during the outbreak was low.

Dengue viruses cause a range of clinical illness, including dengue fever, dengue hemorrhagic fever, and dengue shock syndrome. Dengue is transmitted by infected *Aedes* mosquitoes and is endemic in many areas in the world (1). The occurrence of dengue is rising worldwide, particularly in the Americas, where the reported incidence more than tripled from 1996 to 2002 (2). At the beginning of the 21st century, dengue fever and dengue hemorrhagic fever are the most important arboviral diseases of humans, with an estimated 50–100 million dengue fever cases and several hundred thousand dengue hemorrhagic fever cases occurring each year (3).

In September 2001, the Hawaii Department of Health (HDOH) identified the first autochthonous case of dengue fever in 56 years. The ensuing dengue virus 1 (DENV-1) outbreak, transmitted by *Aedes albopictus* mosquitoes, spanned 9 months. A total of 122 laboratory-positive infections cases were identified from 3 islands: Maui (n = 92), Oahu (n = 26, and Kauai (n = 4) (Figure 1). Seven (6%) of the dengue infections were documented among nonresidents, all of whom had stayed in the Hana area of Maui.

**Figure 1:**
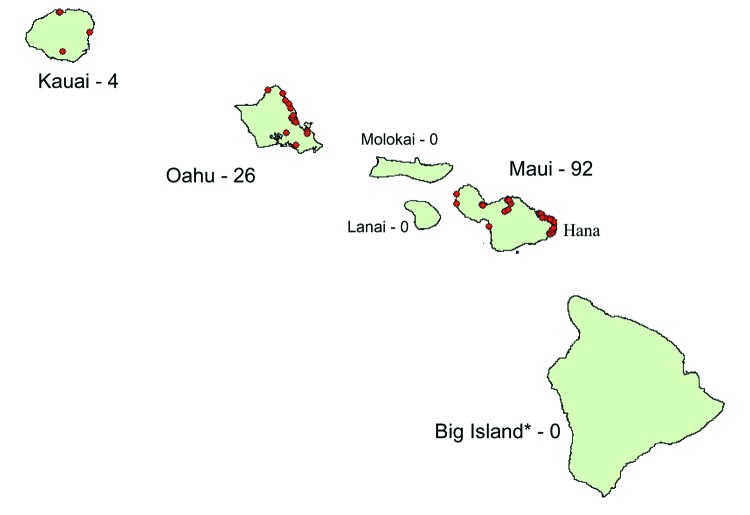
Hawaiian Islands. Areas with dengue activity during the 2001–2002 outbreak are marked in red; the number of laboratory-positive cases is noted adjacent to the island name. *The island of Hawaii is usually called Big Island to avoid confusion with the state of Hawaii.

Despite the relatively high rates of dengue in many tropical destinations frequented by tourists and numerous case reports of dengue in travelers, limited information is available on the risk for infection among short-term visitors (4–8). An estimate of 1 case of dengue illness per 1,000 travelers has been reported recently; however, this figure was derived from surveys of soldiers and expatriates living abroad, i.e., persons whose exposure risks may be quite different from those of recreational travelers (9). Given that Hawaii receives >7 million visitors each year, even low rates of dengue transmission to visitors during an outbreak could result in substantial numbers of infections being exported to the US mainland and elsewhere. We studied the risk for dengue infection among visitors to Hawaii during the peak of the outbreak in 2001.

## Methods

**Figure 2:**
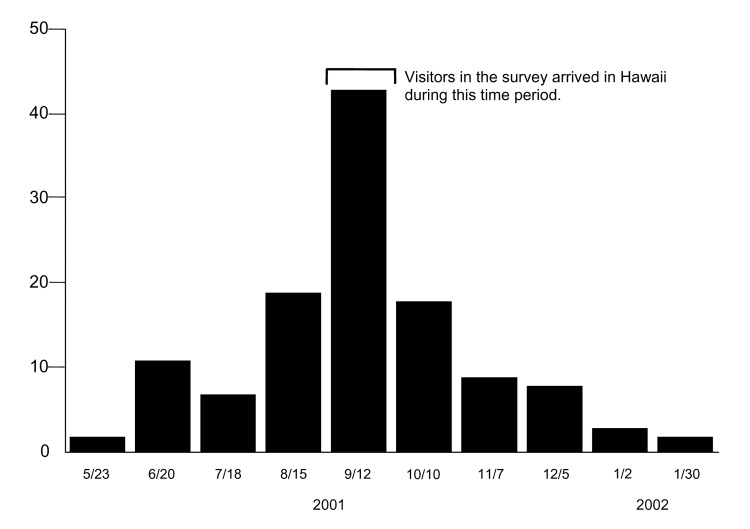
Date if illness onset for 122 laboratory-positive dengue infections, by 4-week period, Hawaii, May 23, 2001–January 30, 2002.

State law requires passengers and families to submit a "Plants and Animals Declaration Form" on arrival in Hawaii. The declaration also solicits information regarding the nature of the passengers' visit to the state, including proposed destinations and intended length of stay. After review at the port of entry, the declaration forms are forwarded to Department of Business and Economic Development and Tourism for data entry and storage. We selected the 4 weeks for which the incidence of illness onset for laboratory-positive dengue infections in Hawaii was the highest (September 12–October 10, 2001) and identified 99,766 parties with a declaration who arrived in Hawaii during that time frame (Figure 2). The person who completed the form was defined as the "visitor" for our study. To be eligible for inclusion, visitors had to 1) supply a complete mailing address within the United States, excluding Hawaii; 2) be visiting Hawaii, as opposed to an in-transit layover (<24 hours) to another destination; and 3) express an intention to visit just 1 of the following islands during their stay—Maui, Oahu, or Hawaii. By sampling visitors who intended to visit only 1 island, we hoped to reduce the number of respondents reporting multiple exposures so that any dengue infections identified could be attributed to a specific island.

A total of 43,161 (43%) visitors fit the criteria. From these, a stratified, random sample of 4,000 visitors was selected: 2,000 visitors to Maui and 1,000 visitors each to Oahu and Hawaii. (The island of Hawaii is commonly called the Big Island and is referred to as such for the remainder of this article to avoid confusion with the state of Hawaii.) Because most dengue infections identified during the outbreak were in residents of Maui, visitors to this island were oversampled to increase the probability of detecting dengue infections among travelers. In contrast, visitors to the Big Island were used as a comparison group because no dengue infections had been identified from exposures on that island during the outbreak (Figure 1).

On December 11, 2001, a questionnaire packet was mailed to the 4,000 visitors in our sample. The packet contained a letter explaining the purpose of the study, a self-addressed stamped envelope, and a 14-question survey. Visitors were asked demographic information, dates of travel to Hawaii, knowledge of the dengue outbreak, changes made to vacation plans due to the outbreak, actions taken to reduce mosquito exposures, which islands were visited, and specifically whether the visitor had traveled to the Hana, Maui, area. In addition, the questionnaire asked if the visitor became ill during or up to 14 days after the trip, and if so, what symptoms were experienced. Finally, visitors were asked to provide a name, phone number, or email address so they could be contacted by HDOH.

Denguelike-illness (DLI) was defined as fever and/or chills with at least 1 additional symptom, including the following: headache, body aches, eye pain, muscle aches, joint pain, rash, bleeding gums, blood in the stool, or nosebleed. Our description of clinical DLI was intentionally less restrictive than that used for surveillance by the World Health Organization (WHO), which uses fever plus 2 other compatible symptoms. We also allowed a history of chills to serve as an indicator of fever when no temperature was taken because we wanted to increase the probability of identifying any dengue infections that had occurred in visitors (1,10).

Visitors reporting DLI were contacted. After a brief description of the outbreak, and risks and benefits of participating, they were asked to consent to having blood drawn for serologic testing. HDOH arranged to have blood drawn in conjunction with the visitor's local health department, doctor, or diagnostic laboratory at no cost to the patient. All specimens were tested for anti-dengue immunoglobulin (Ig) G antibodies at the Division of Vector-Borne Infectious Diseases, Centers for Diseases Control and Prevention (CDC), by methods previously described (11,12).

### Statistical Evaluation

Statistical analyses were performed by using SAS (version 8.2, Cary, NC, USA) and SAS-Callable SUDAAN release 8.0 (Research Triangle Institute, Research Triangle Park, NC, USA). Responses were weighted to reflect the visitor's port-of-entry (which island they originally flew into from the mainland) and their intended island destination; the survey weights were created by SMS Research and Marketing Services, Inc (Honolulu, HI, USA). In the following text, numbers are presented unadjusted, followed by the weighted percentage unless otherwise noted; chi-square and relative risks are weighted.

The incidence of dengue infection among participants was calculated as the number of visitors who were anti-dengue IgG positive, divided by the total number of person-days in Hawaii for all those serologically tested. The upper 95% confidence limit (CL) for dengue incidence was calculated on the basis of the Poisson distribution as Maximum risk = –ln (0.05)/*t* x (365), where *t* is the number of person days of exposure (13,14). The state of Hawaii's HDOH Institutional Review Board approved the study through expedited review on March 12, 2002.

## Results

**Figure 3:**
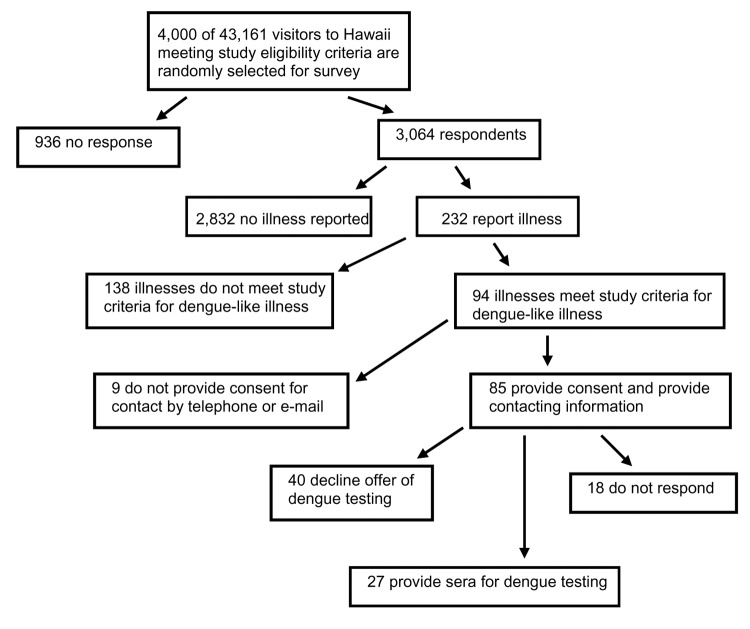
Participation rates in the survey and serologic testing to assess the risk for dengue transmission to visitors during an outbreak in Hawaii, 2001. See text for further details.

A total of 3,064 (77%) visitors responded to the survey (Figure 3). Respondents were 47% male and 53% female. The median age was 44 years (range 14–94 years). Respondents were similar to nonrespondents in terms of sex and the number of days spent in Hawaii. Most respondents and nonrespondents were 30–59 years of age (69% and 67%, respectively).

**Figure 4:**
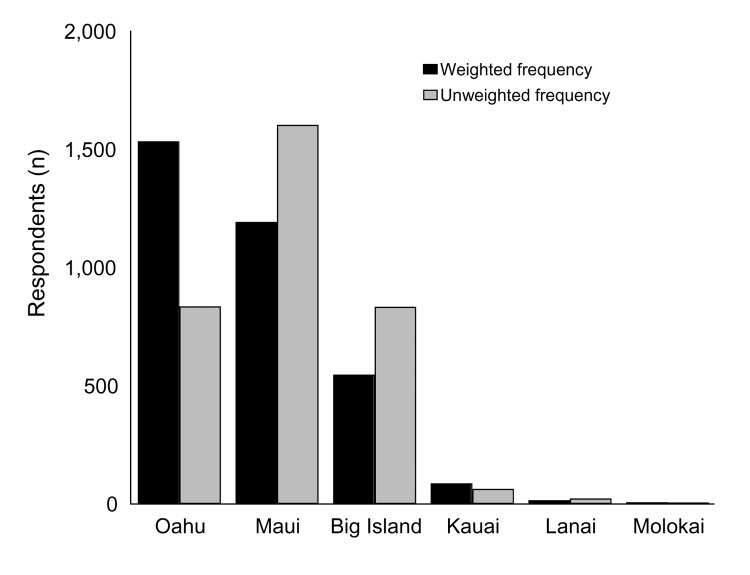
Island visited for 3,064 survey respondents, by weighted and unweighted frequencies. Big Island is the term used for the island of Hawaii to avoid confusion with the state of Hawaii. More than 1 island could be listed for each respondent, but most visitors went to only 1 island, that is, 3,384 island visits were reported by 3,064 respondents.

The median length of stay in Hawaii was 8 days (range 2–188 days). Resort hotels were the most common accommodation (60%), followed by condominiums (22%), private residences (16%), bed and breakfast hotels (1%), and campgrounds (0.4%). The distribution of island destinations among respondents is shown in Figure 4. A total of 2,729 (88%) of the respondents visited only the island they had indicated on the agricultural declaration upon arrival in Hawaii; 94 (4%) went to the island indicated on the declaration form as well as to other islands; and 241 (8%) visited an island(s) other than the one indicated on the declaration. Sixteen percent of all respondents (n = 655) reported going to Hana, Maui, of whom 74 (12%) stayed overnight.

### Knowledge of the Outbreak

**Figure 5:**
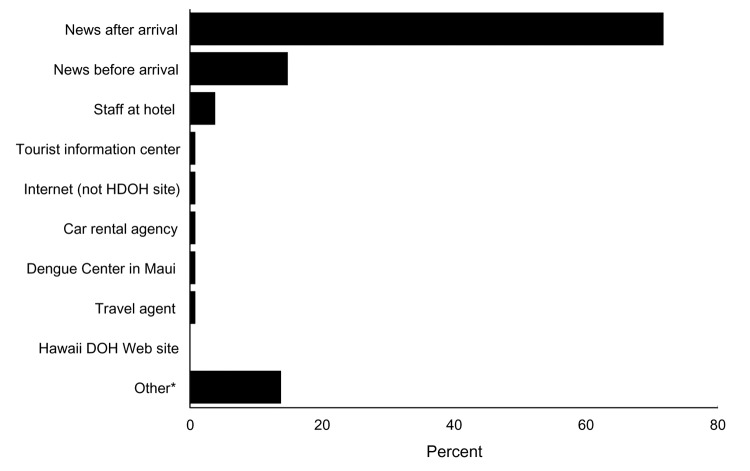
Source of information through which 1,949 visitors to Hawaii first learned of the dengue outbreak in 2001. HDOH, Hawaii Department of Health. *Other sources of information included family, friends, or co-workers who learned of the outbreak from the media or Internet; signs posted in and around Hana, Maui; and other tourists or Hawaii residents who informed the respondent.

Only 238 (8%) of respondents reported that they knew about the dengue outbreak before they traveled to Hawaii; 1,467 (45%) learned of it while in Hawaii; 244 (9%) first heard of it after leaving Hawaii but before receiving the survey questionnaire, and 1,071 (37%) learned of the outbreak from the HDOH survey. Of the 1,949 (62%) visitors who knew of the outbreak before receiving the questionnaire packet, >80% first learned of the outbreak from the news media (newspaper, radio, or television) (Figure 5).

Of the 1,705 respondents who learned of the outbreak either before or during their visit to Hawaii, 483 (27%) took personal precautions to reduce their exposure to mosquito bites, and 213 (10%) changed their vacation plans because of the outbreak. Among those who changed their plans, 146 (66%) did not travel to Hana, Maui; 73 (35%) skipped activities such as camping, hiking, or golfing that they had originally planned; 9 (5%) changed the types of places they stayed at while in Hawaii; 5 (5%) decided not to travel to Maui; and 7 (5%) shortened their vacation to Hawaii. Determining how many deferred coming to Hawaii because of the dengue outbreak, of course, is not possible. Of those who took personal precautions to avoid mosquito bites, 332 (66%) used repellent, 118 (24%) left places if mosquitoes were seen, 83 (18%) limited their time outdoors, 80 (17%) wore long pants and long-sleeved shirts when outdoors, and 38 (9%) used mosquito coils, sprayed insecticide in or around the places that they stayed, or both.

A total of 232 (8%) visitors reported becoming ill either during their trip or within 14 days of leaving Hawaii. The reported illness in 138 (59%) of the ill respondents did not meet criteria for DLI (Table). Respondents with illness that did not meet DLI criteria reported a median of 2 symptoms; headache, nausea, and diarrhea were the most frequently reported among this group.

**Table:** Symptoms reported by 232 visitors after travel to Hawaii, September 12–October 10, 2001*

Symptom	Reported (% frequency) by participants with DLI, n = 94	Reported (% frequency) by participants without DLI, n = 138	Chi-square†
Chills	80 (88)	7 (3)	114.0†
Headache	74 (79)	58 (47)	17.8†
Body pain	59 (62)	29 (18)	32.1†
Muscle aches	58 (64)	27 (19)	32.2†
Fever	58 (56)	7 (4)	51.2†
Extreme tiredness	51 (49)	34 (22)	13.4†
Nausea	44 (45)	31 (25)	6.9†
Diarrhea	38 (37)	31 (25)	2.4
Joint pain	28 (31)	20 (14)	6.9†
Vomiting	23 (20)	18 (15)	0.7
Pain behind the eyes	22 (29)	16 (12)	6.6†
Itching	15 (14)	10 (8)	1.5
Rash	13 (13)	18 (13)	0.0
Shortness of breath	10 (15)	7 (7)	2.1
Dark stool	7 (7)	4 (3)	1.2
Bloody nose	4 (7)	3 (3)	1.1
Bleeding gums	4 (4)	0	2.9

*DLI, denguelike illness, was defined as fever and/or chills, plus any of the following symptoms: headache, body aches, eye pain, muscle aches, joint pain, rash, bleeding gums, melena (dark stools), or nosebleed. Figures are exact; percentages and chi-square values were calculated by using survey weights. †p<0.05.

Ninety-four visitors (3% of all respondents and 43% of those ill) met criteria for DLI, of which 12 reported at least 1 hemorrhagic manifestation. Respondents with DLI reported a median of 7 symptoms; chills, headache, body pain, muscle aches, and fever were each reported by more than half of respondents with DLI.

Of visitors with DLI, 38 (40%) became ill while still vacationing in Hawaii, 34 (36%) became ill 1–10 days after leaving Hawaii and 16 (18%) became ill 11–14 days after leaving the islands. The median duration of illness was 7 days (range 1–60). Thirty-four (34%) of the visitors with DLI saw a doctor for their illness; in 2 patients, dengue was diagnosed by their physician, and 2 others stated that they had been hospitalized, once for suspected meningitis and once for a respiratory infection.

The proportion of visitors reporting any illness (7%–8%) or DLI (3%) was very similar for each of the 3 islands (Oahu, Maui, and the Big Island). Visiting the epicenter of outbreak, Hana, Maui, was not significantly associated with reporting DLI. However, staying overnight in Hana was associated with reporting DLI symptoms when compared to visitors without illness (relative risk = 2.78, 95% CI 1.04–7.43). The type of accommodation used in Hawaii (hotel, condominium, bed and breakfast) was not significantly associated with reporting DLI.

Nine (9%) of the 94 persons with DLI did not provide a telephone number or email address for follow-up. Of the remaining 85 (91%) respondents with DLI, 27 (32%) provided a serum specimen for testing through their local department of health or physician; 18 (23%) could not be contacted after repeated attempts; and 40 (45%) refused testing or did not show up for their scheduled appointment to draw blood.

The 27 visitors who had blood drawn stayed in Hawaii for a median of 8 days (mean 13 days). The median interval from illness onset to collection of the serum was 224 days (range 153–310). All samples from the 27 persons were negative for anti-dengue IgG antibodies. These 27 visitors contributed a total of 358 person-days of exposure during the outbreak; the point estimate of dengue incidence is therefore zero infections per 358 person-days of exposure (0 per person-year) with an upper 95% CL of 3.0 cases per person-year (unweighted data).

## Discussion

The state of Hawaii currently receives 5.3 million domestic and 2.2 million international visitors each year. In light of this, the recent reemergence of dengue fever in Hawaii represents a potential threat to populations across the US mainland and elsewhere. The current study was undertaken to assess the risk of visitors acquiring dengue infection during the outbreak. This information is needed because of the sheer number of visitors and the high likelihood that dengue will be reintroduced into Hawaii in the future.

Our investigation indicated that the risk for dengue among domestic travelers to Hawaii during the peak of the outbreak was low. Although 3% of the visitors surveyed reported experiencing DLI, none of the persons who underwent anti-dengue IgG antibody testing had evidence of dengue infection. The major limitation of this study is that only approximately one quarter of persons with DLI provided a serum specimen. However, these persons were similar to those with DLI who did not provide a specimen in regard to age, sex, islands visited, and number of days spent in Hawaii. In addition, the median number of symptoms reported by those with DLI who provided a blood specimen and those who did not was the same for both groups, and no significant differences were found between groups with regard to individually reported symptoms. In aggregate, these data suggest that the results from the subset who provided a specimen for testing may be representative of all who reported DLI.

Two additional pieces of information corroborate our assessment that the risk for dengue to travelers in this outbreak was low. First, we found that the proportion of travelers reporting DLI was the same for visitors to each of 3 islands, Maui, Oahu, and the Big Island. This finding is in stark contrast to the incidence of dengue infection among Hawaii residents during the outbreak, where the rates per 100,000 persons were dramatically different, 73, 7, and 0 for Maui, Oahu, and the Big Island, respectively. If DLI were a specific indicator of dengue infection, one might have expected the proportion of visitors reporting DLI by island to roughly parallel the risk profile observed for island residents. In other words, the lack of concordance between the trends in dengue incidence rates among residents of the various islands during the outbreak and the proportion of visitors with DLI suggests that the reported DLI illnesses, including those not evaluated serologically, were not dengue fever.

Second, during the outbreak investigation conducted before this survey, we made a concerted attempt to identify travelers with dengue with little success. These efforts included a request to all state epidemiologists to consider dengue infection among persons returning ill from Hawaii, communication through CDC's Epi-X system, and extensive outreach through venues frequented by visitors (car rental agencies, hotels, tourist information centers). Only 7 (6%) of the 122 persons with laboratory-positive dengue infection were nonresidents. All 7 nonresidents with dengue stayed at rental homes in the epicenter of the outbreak (Hana, Maui), and 6 of the 7 had occupied the same house. Another 70 nonresidents who visited Hawaii during the outbreak were reported to HDOH by other states as having possible dengue infections; 30 agreed to be serologically tested—all results were negative.

The low rate of dengue infections is difficult to attribute to personal protective measures undertaken by the travelers. More than 40% of the visitors surveyed did not know about the outbreak until after their vacation had ended. Of the ≈60% who learned of the outbreak before arriving in Hawaii or while on vacation, only 26% took personal precautions to avoid mosquito bites (e.g., using mosquito repellent) and just 10% changed their activities to reduce potential exposures.

While imperfect, the most effective way of alerting visitors about the outbreak appears to be through the media, including radio, television, and newspapers. Among visitors who learned of the outbreak before or during their visit, almost 90% stated that they first heard about it through these channels. Other sources of information such as travel agents, Web pages, hotel staff, car rental agencies, and information centers appear to have been less informed, but they can still play an important role in providing important supplemental information. Since many people on vacation purposefully avoid television and newspapers, new strategies should be explored for increasing the proportion of travelers who can be reached with important health protection messages if needed in the future.

We caution that the low risk for dengue infection among travelers to Hawaii may not be applicable to other settings where dengue is endemic, where the predominant mosquito vector is *Ae. aegypti*, or when outbreak-associated attack rates are much higher than that observed in Hawaii during 2001 to 2002. However, relatively few studies have attempted to quantify the risk for dengue infection among short-term travelers, and the data available from other cohorts encompass a wide range. One cohort study in Puerto Rico identified no recent dengue infections in 153 relief workers, and the investigators estimated the upper limit of risk to be 1.7 dengue infections per person-year exposure (13). A study of Swedish travelers estimated the risk for dengue fever to be highest among visitors to the Indian subcontinent and the Malay Peninsula (30–58 infections per 100,000 travelers) (15). However, another study among Israeli travelers to Southeast Asia estimated the infection rate to be nearly 1,000-fold higher (3–5 per 1,000 travelers) (16). Moreover, a seroconversion rate of 6.7% was reported among 104 younger Israeli travelers on extended trips (3–16 months) to the tropics (8). The lack of homogeneity of these data suggests that factors such as location of travel, the intensity of dengue activity at the time, the length of stay, and the type of travel engaged in are likely to be important determinants of the risk for dengue among visitors in any particular setting.

While a precise estimate of the risk may not be obtainable, >500 laboratory-confirmed and 2,000 suspected dengue infections reported in returning US travelers from 1986 to 2000 indicate that the risk for infection among visitors to dengue-affected areas is not insignificant (17–22). In light of this, we recommend that US clinicians consider the possibility of dengue transmission when evaluating febrile rash illnesses among travelers to areas with a history of dengue activity, including the Pacific Islands.

This study has several limitations. First, as noted above, only approximately one fourth of all persons reporting DLI underwent serologic testing. The most frequent reasons for declining to have blood drawn were inconvenience, an aversion to needles, or sense that since the illness was in the past (on average, 7 months before) and had resolved, testing had little benefit. If we were to conduct this type of study in the future, we would make a concerted attempt to shorten the time between departure from Hawaii and follow-up contact.

A second limitation is that we sought to test only persons who had symptoms compatible with dengue fever; therefore, our efforts would not have identified infections that were very atypical in presentation or asymptomatic. However, we used a fairly inclusive definition of DLI, and there is no reason to believe the rate of dengue infection would have been appreciably higher among those who were asymptomatic as compared to those with compatible symptoms. Third, illness and exposure histories were self-reported and therefore potentially subject to recall bias.

In summary, our findings suggest that the risk for dengue for travelers to Hawaii during the 2001–2002 outbreak was limited. Given Hawaii's interconnectedness to areas of the world with endemic and epidemic dengue, Hawaii will likely experience another outbreak in the future. Should that occur, the most effective means currently available for informing visitors is through the media; the message to tourists should be that the risk for dengue infection is likely to be low, but it still exists.
